# Dynamic modeling of practice effects across the healthy aging-Alzheimer’s disease continuum

**DOI:** 10.3389/fnagi.2022.911559

**Published:** 2022-07-28

**Authors:** Andrew R. Bender, Arkaprabha Ganguli, Melinda Meiring, Benjamin M. Hampstead, Charles C. Driver

**Affiliations:** ^1^Department of Epidemiology and Biostatistics, College of Human Medicine, Michigan State University, East Lansing, MI, United States; ^2^Graduate Program in Neuroscience, College of Natural Science, Michigan State University, East Lansing, MI, United States; ^3^Michigan Alzheimer’s Disease Research Center, Ann Arbor, MI, United States; ^4^Department of Statistics and Probability, College of Natural Science, Michigan State University, East Lansing, MI, United States; ^5^Mental Health Service, VA Ann Arbor Healthcare System, Ann Arbor, MI, United States; ^6^Research Program on Cognition and Neuromodulation Based Intervention, Department of Psychiatry, University of Michigan, Ann Arbor, MI, United States; ^7^Institute of Education, University of Zurich, Zurich, Switzerland; ^8^Institute for Educational Evaluation, Associated Institute at the University of Zurich, Zurich, Switzerland

**Keywords:** practice effects, aging, learning, mild cognitive impairment, verbal memory, dementia, dynamic modeling, Alzheimer’s disease (AD)

## Abstract

Standardized tests of learning and memory are sensitive to changes associated with both aging and superimposed neurodegenerative diseases. Unfortunately, repeated behavioral test administration can be confounded by practice effects (PE), which may obscure declines in level of abilities and contribute to misdiagnoses. Growing evidence, however, suggests PE over successive longitudinal measurements may differentially predict cognitive status and risk for progressive decline associated with aging, mild cognitive impairment (MCI), and dementia. Thus, when viewed as a reflection of neurocognitive plasticity, PE may reveal residual abilities that can add to our understanding of age- and disease-related changes in learning and memory. The present study sought to evaluate differences in PE and verbal recall in a clinically characterized aging cohort assessed on multiple occasions over 3 years. Participants included 256 older adults recently diagnosed as cognitively unimpaired (CU; *n* = 126), or with MCI of amnestic (*n* = 65) or non-amnestic MCI (*n* = 2085), and multi-domain amnestic dementia of the Alzheimer’s type (DAT; *n* = 45). We applied a continuous time structural equation modeling (ctsem) approach to verbal recall performance on the Hopkins Verbal Learning Test in order to distinguish PE from individual occasion performance, coupled random changes, age trends, and differing measurement quality. Diagnoses of MCI and dementia were associated with lower recall performance on all trials, reduced PE gain per occasion, and differences in non-linear dynamic parameters. Practice self-feedback is a dynamic measure of the decay or acceleration in PE process changes over longitudinal occasions. As with PE and mean recall, estimated practice self-feedback followed a gradient from positive in CU participants to null in participants with diagnosed MCI and negative for those with dementia diagnoses. Evaluation of sensitivity models showed this pattern of variation in PE was largely unmodified by differences in age, sex, or educational attainment. These results show dynamic modeling of PE from longitudinal performance on standardized learning and memory tests can capture multiple aspects of behavioral changes in MCI and dementia. The present study provides a new perspective for modeling longitudinal change in verbal learning in clinical and cognitive aging research.

## Introduction

Standardized neuropsychological tests are sensitive to cognitive declines associated with older age and incident mild cognitive impairment (MCI) and dementia. Clinical characterization of cognitive impairments and the tracking of progressive declines requires repeated testing, but performance on repeated standardized tests is contaminated by practice effects (PE; Duff et al., 2001; Hawkins et al., 2004; Salthouse, 2010; Hoffman et al., 2011). This contamination arises due to the incidental retention of information from prior exposure to test format and content, which can enhance performance at subsequent reinstatement (Wilson et al., 2000; Heilbronner et al., 2010; Machulda et al., 2013). The potential of PE to mask true cognitive declines in healthy and pathological aging has motivated numerous attempts to remove PE from estimates of level or change in performance (Rabbitt et al., 2001; Salthouse and Tucker-Drob, 2008; Salthouse, 2010; Calamia et al., 2012). However, as a measure of the capacity to benefit from repetition, PE may represent an independent behavioral dimension sensitive to declines in older age and neurodegenerative disease (Yang, 2011; Duff et al., 2012). Thus, rather than treat PE as noise, approaches to integrate modeling of PE and cognition may provide novel clinical value in characterizing cognitive impairment and dementia.

Notably, simulation study findings show PE are not easily distinguished from true changes associated with aging or cohort effects (Hoffman et al., 2011). Therefore, quantifying PE as the change dimension of interest may better serve short-term characterization of functional declines in MCI and dementia. This proposition is in accord with suggestions that variation in PE reflects individual differences in neurocognitive plasticity (Baltes and Raykov, 1996; Yang and Krampe, 2009; Yang, 2011). Others have reported PE as a marker of clinical declines in older adults with mild cognitive impairment (MCI) or dementia of the Alzheimer’s type (DAT; Duff et al., 2007, 2012; Fernandez-Ballesteros et al., 2012; Sanchez-Benavides et al., 2016). Lower PE is also associated with performance decrements in cognitively intact adults with preclinical Alzheimer’s pathology (Goldberg et al., 2015; Hassenstab et al., 2015). These findings highlight the intrinsic dependencies between the contributions of prior experience to cognitive performance and vulnerability to decline.

Verbal learning tasks provide established clinical markers of neuropsychological deficits associated with diagnoses of MCI and DAT (Duff et al., 2001; Hawkins et al., 2004; Blasi et al., 2009; Lonie et al., 2010; Summers and Saunders, 2012). Standardized tests of verbal learning and memory typically involve serial auditory presentation of lists of verbal stimuli, immediately followed by instructions to freely recall all words remembered. Most standardized tasks then repeat this procedure for multiple trials with the same stimuli, followed by a delay and an additional free recall trial. Due to their repetitive nature, verbal learning tasks are particularly vulnerable to PE when content is repeated across longitudinal administrations (Duff et al., 2001; Heilbronner et al., 2010; Machulda et al., 2013; Campos-Magdaleno et al., 2017). Arguably, repeated free recall performance on multiple trials distributed over longitudinal occasions embodies the definition of a dynamic process – i.e., one that constantly changes and progresses (Zimprich et al., 2008). Moreover, serial recall represents retrieval-based learning, in which retrieval of a representation updates the representation itself (Karpicke et al., 2014). Furthermore, each repeated trial involves not just encoding and retrieval, but updating and retrieval monitoring, as well as potential metacognitive processes (Hertzog and Dunlosky, 2004; Bender and Raz, 2012). Thus, successful recall performance involves multiple interactive executive processes, which may also show decrements in the presence of phenotypic cognitive impairment. Yet, the extent that task summary scores reflect these dynamics is unclear.

To date, there is neither consistent operationalization nor definition of PE in the contexts of clinical and basic cognitive aging research. Studies report PE estimated both in variable time scales ranging from minutes to years and from a host of different behavioral tasks, conditions, and stimuli. In addition, PE is largely quantified in extant studies of normative and pathological aging using difference scores or *via* linear modeling frameworks (Raykov et al., 2002; Salthouse et al., 2004; Duff et al., 2007; Bender et al., 2013, 2020; Goldberg et al., 2015; Hassenstab et al., 2015). However, linear modeling approaches may fail to capture the interactive, dynamic processes involved in longitudinal verbal learning task performance. Modern dynamic modeling methods that can quantify non-linear processes may provide novel markers of PE or cognitive decline. In the context of longitudinal changes in verbal recall, dynamic modeling can account for the current level of performance at each trial and occasion to help predict subsequent performance. Thus, within-occasion and longitudinal performance are modeled as interdependent processes that play out over time. Modeling performance on each verbal recall trial as an individual interactive process, manifest over multiple occasions, permits estimating PE as a change process independent of overall mean performance and trial-by-trial random effects.

The continuous time structural equation modeling (*ctsem*; Driver et al., 2017) framework applies a differential equations-based time series analysis for modeling ongoing dynamic processes, coupled with a measurement layer to delineate measurement noise from true change. While it resembles latent growth and latent change score models, *ctsem* permits treating time-in-study as a continuous variable, in addition to other key enhancements. Relevant to longitudinal verbal learning performance, the framework permits specifying a non-linear measurement model to account for factors such as differential measurement error across groups or levels of performance. It also allows modeling random effects to capture individual differences in all system parameters, as well as covariates that can predict such individual differences. Furthermore, these non-linear processes may also be sensitive to phenotypic cognitive impairment, possibly independent of level of performance or PE. Thus, dynamic modeling of longitudinal verbal learning data to decompose PE from trial-level performance may offer additional value for clinical aging populations previously reported to show a loss of PE.

Extant findings show diagnoses of MCI and multi-domain amnestic dementia (DAT) are associated with reduced or non-existent PE on standardized verbal learning tasks (Duff et al., 2007, 2019; Calamia et al., 2012; Goldberg et al., 2015; Gavett et al., 2016). However, it is unclear whether differences in PE associated with MCI or dementia are also influenced by other factors known to influence verbal memory. For example, whereas older age is associated with performance decrements on episodic memory tasks, female sex is associated with better verbal episodic memory (Herlitz et al., 1997; Herlitz and Rehnman, 2008; Bender et al., 2010). Furthermore, greater educational attainment also confers a higher initial level of premorbid performance on memory tasks (Lovden et al., 2020). Still, it is unclear if such individual differences may modify the larger effects of MCI or DAT diagnosis on verbal recall or PE, particularly over less expansive periods of assessment.

The University of Michigan Memory and Aging Project (UM-MAP) includes older participants clinically characterized as cognitively unimpaired (CU) or diagnosed with MCI or DAT. The available data includes one to four occasions of annual neuropsychological assessment, including the Hopkins Verbal Learning Test (HVLT), which was administered using the same stimulus lists on each occasion of measurement. This provided an opportunity to apply *ctsem* for modeling longitudinal verbal recall performance and PE as dynamic processes in a clinical aging sample. To our knowledge, this is the first attempt to apply dynamic modeling to quantify longitudinal changes in verbal learning, particularly in a clinical aging context. Critically, dynamically modeled estimates of PE in the present study served as the primary measure of longitudinal change in performance, rather than estimating change in ability and PE separately. We hypothesized that both older age and diagnosed MCI and DAT would be associated with poorer recall and lower PE. We also expected the effects of clinical diagnosis would be modified by individual differences in chronological age, sex, and educational attainment. Specifically, we hypothesized that higher education and female sex would be associated with better verbal recall; however, we had no clear expectations regarding how these would influence effects of MCI or DAT diagnosis on recall or PE.

## Materials and methods

### Participants

The study sample was drawn from research participants in the University of Michigan Memory and Aging Project (UM-MAP), which is the primary clinical cohort at the Michigan Alzheimer’s Disease Research Center (MADRC). The sample included 256 participants (67% women) from 51 to 89 years of age at the first assessment. At each measurement occasion all participants underwent neuropsychological evaluation and a consensus diagnosis was made during a consensus conference by neurologists, neuropsychologists, nurses, social workers or other specialists as appropriate using the National Alzheimer’s Coordinating Center (NACC) criteria. The sample was divided into three subgroups based on the last recorded diagnosis for each participant (Table 1): cognitively unimpaired (CU; *n* = 126; 71% women), amnestic or non-amnestic MCI (MCI; *n* = 85; 67% women) and multi-domain amnestic dementia (DAT; *n* = 45; 60% women) consistent with Alzheimer’s disease and mixed dementia. Over the course of the study, six participants progressed from diagnoses of aMCI to DAT of the Alzheimer’s type, and an additional six participants changed from CU to MCI diagnoses. In contrast, one participant initially diagnosed with DAT was subsequently characterized as CU, and 15 participants characterized with MCI at baseline reverted to CU at their final study assessment 2–3 years later.

**TABLE 1:** Participant characteristics by clinical diagnosis.

	CU	MCI	DAT
	Mean (*sd*)	Mean (*sd*)	Mean (*sd*)
Age	70.06 (6.43)	72.66 (8.04)	72.22 (9.31)
Education	15.90 (2.67)	15.60 (2.47)	15.51 (2.61)
Systolic BP	134.43 (22.97)	139.46 (22.10)	133.81 (15.67)
Diastolic BP	77.88 (11.35)	81.10 (12.03)	74.91 (9.76)
CDR	0.33 (0.41)	0.97 (0.75)	3.67 (2.08)
MoCA	26.85 (1.93)	23.03 (3.19)	15.45 (5.64)
GDS	1.13 (1.39)	1.43 (1.70)	1.60 (1.33)

Values are mean with standard deviation in parentheses. CU, cognitively unimpaired; MCI, diagnosis of amnestic or non-amnestic MCI; DAT, diagnosis of multi-domain amnestic dementia. Age and educational attainment are in years. Systolic and diastolic BP are blood pressure measured in mmHg. CDR, clinical dementia rating; MoCA, montreal cognitive assessment. GDS, geriatric depression scale.

### Longitudinal organization

Following baseline assessment participants returned annually for repeated testing. The present study data included assessments on one to four separate measurement occasions (Table 2), separated by mean intervals of 1.09 years. Mean intervals between each occasion of measurement, separately by subgroup are reported in Supplementary Table 1.

**TABLE 2:** Participant counts for total number of measurement occasions by clinical diagnosis.

Clinical diagnosis	Total number of occasions				
	**1**	**2**	**3**	**4**	**Total**
CU	20	45	40	21	126
MCI	34	23	20	8	85
DAT	20	19	6	0	45
Total	74	87	66	29	256

CU, cognitively unimpaired; MCI, diagnosis of amnestic or non-amnestic MCI; DAT, diagnosis of multi-domain amnestic dementia. Values represent counts of participants by their total number of measurement occasions. For example, in the top row for CU participants, 20 had HVLT data for only one occasion, 45 participants completed two occasions, 40 had three occasions of data, and 21 CU participants had complete data for all four occasions.

### Cognitive testing

All participants were administered the Hopkins Verbal Learning Test (HVLT; Brandt, 1991) on each occasion of measurement and testing followed the published procedures. The HVLT auditorily presents 12-item lists of semantically linked verbal stimuli, presented at a rate of 2 s per item. Following presentation of all items, the participant freely recalls as many as possible. The score per trial is the total number of correctly recalled words. This is repeated for two additional free recall trials, using the same verbal stimuli. A 20-min delay follows the third recall trial, after which participants are asked to freely recall as many words as possible without re-presenting the stimuli. Notably, although the HVLT also includes additional delayed recall and recognition measures, the present study focused on the first four trials, i.e., the three immediate and first delayed recall trials. Critically, the present study repeated the same lists of verbal stimuli across all occasions of measurement.

### Data analysis

To analyze performance, change as a function of PE, and individual differences therein, we developed hierarchical Bayesian continuous time dynamic models (Driver et al., 2017) implemented in the *ctsem* software (Driver et al., 2017;Driver and Voelkle, 2021). A more detailed description of the model and corresponding mathematical apparatus follows below in the Supplementary Material.

#### Modeling practice effects and performance in *ctsem*

To account for varying observation timing and to allow for continuously interacting processes, *ctsem* estimates an underlying continuous-time model, which is translated into discrete time expectations and covariance matrices using matrix exponentiation (Voelkle et al., 2012; Voelkle and Oud, 2013). To account for the multiple timescales at play (i.e., within and between occasion), each of the immediate (i.e., Trials 1, 2, and 3) and delayed recall trials (Trial 4) were modeled as independent latent processes over four occasions of measurement, with correlated random disturbances. This means that although we may not have been able to predict every fluctuation in performance, when an unpredicted fluctuation occurs this contributes to predictions for the other trials within and (potentially) across occasion. We estimated the standard deviation and within-occasion correlations of the diffusion process, separately for each Trial (e.g., Diffusion T1). These parameters capture the extent of unpredictable random changes across measurement occasions, which are nevertheless useful for predicting performance on other trials within-occasion, or across-occasion – thus more likely representing some genuine aspect of performance. In contrast, the standard deviation of the measurement error (i.e., *measurement error*) captures unpredictable changes in observed performance that do not provide value for prediction on other trials. The model also contained a parameter reflecting *Trial self-feedback* (*sf_Trial*)*;* this parameter describes the persistence of the random changes between measurement occasions for each trial. Put differently, sf_Trial represents the extent to which unpredicted shifts up or down (independent of measurement error) on performance for a specific trial, can be used to predict performance for the same trial number on the next occasion, i.e., across-occasion persistence. On top of this base structure allowing for correlated random processes, PE was modeled as a latent process that changed at the end of each occasion. As for the trial specific processes, we also specified a Practice self-feedback (sf_Practice) parameter to provide an estimate of total feedback on PE; this parameter serves as a measure of the decay or acceleration in the change to Practice effect process over the observed range of occasions. Like sf_Trial, the sf_Practice parameter reflects the extent that the current level of practice (i.e., at the end of each occasion) contributes to the Practice effect at the next occasion. Thus, a positive sf_Practice value would reflect an increase in gains due to practice on later occasions, whereas a negative sf_Practice value reflects a decay or deceleration of learning processes that reduce total PE and implies reducing gains due to further practice. Each model output includes estimates of population means for all modeled parameters, correlations between trial manifest means and the PE parameter, and estimates of time independent predictor effects and interactions.

#### Time independent predictors and covariates

Parameters of the system and measurement models also varied on an individual level as a function of clinical diagnosis, as well as random effects. The effects of other sources of individual differences were examined in separate sensitivity models to evaluate effect modification by individual linear covariates, including baseline age, sex, and educational attainment. This accounts for a broad range of phenomena, such as heterogeneity of measurement error variance with age and performance. Therefore, we first evaluated most recent clinical diagnosis of MCI or DAT as time independent predictors, in relation to CU participants. This was followed by independent subsidiary sensitivity models that evaluated covariate effects age, sex, and educational attainment (scaled and centered at the respective sample means) on model parameters and their interactions with diagnostic group. Last, independently for the three diagnostic groups we evaluated each of the time independent predictors age, sex, and education in separate models.

#### Bayesian estimation

Due to the large number of parameters and random effects, we opted for Bayesian maximum *a posteriori* estimation. Priors on the parameter means were relatively broad and non-influential, while tighter priors (i.e., pushing estimates toward zero) were used for modeling individual differences to mitigate over-fitting. Despite yielding more conservative estimates, this permits a more pragmatic approach for estimating and interpreting models with many parameters and modest sample sizes. For details on priors, and the expanded stochastic differential equation and related measurement model see the Supplementary Material.

## Results

### A guide to interpreting model results and figures

**FIGURE 1:**
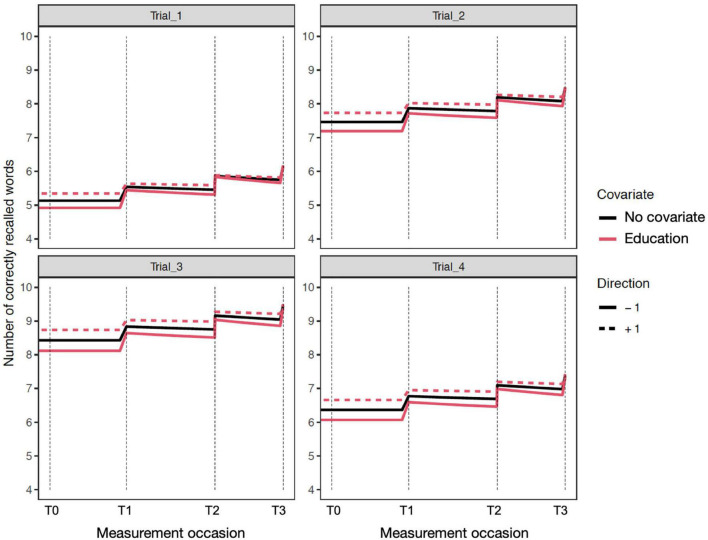
Expectation plots for change in recall performance (black line) across four measurement occasions (e.g., T0: baseline, T1: 1 year). The solid and dashed red lines depict effect modification by education; higher (dashed) and lower (solid) levels of education predict different levels and patterns of change. The complete guide to interpreting expectation plots can be found in Section “A Guide to Interpreting Model Results and Figures.”

**FIGURE 2:**
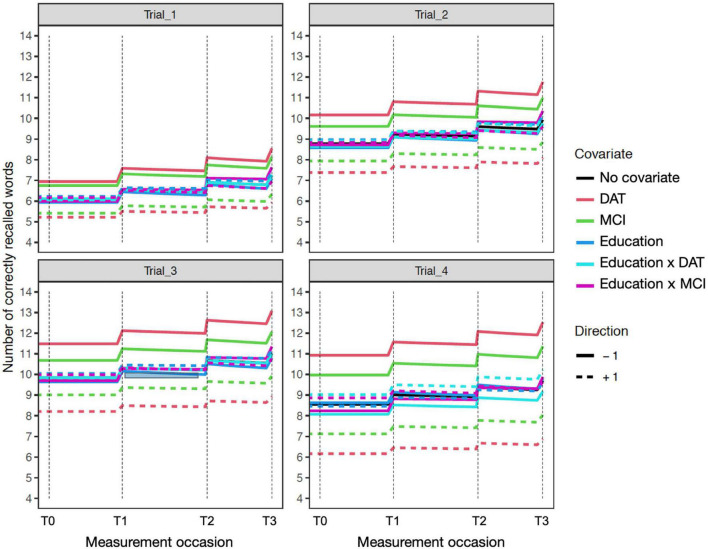
Education sensitivity model expectation plots for change in recall performance (black line) across four measurement occasions (e.g., T0: baseline, T1: 1 year). The solid and dashed colored lines depict effect modification by time independent predictors: DAT diagnosis (red lines), MCI diagnosis (green lines), Education (blue lines; higher: +1; lower: –1), and interactions of Education × DAT and Education × MCI. For covariate effects, higher (dashed) and lower (solid) levels of the covariate are shown to modify the level and expected slope. All covariate effects are in reference to 0 values of other covariates. The dashed red line reflects DAT, and the solid red line represents all other participants. Interaction effects only represent the total additive value of the interaction holding the main effects at zero. For example, in Trial_4 (lower right), the interaction of educational attainment and DAT shows higher education (+1) is associated with better performance in those with dementia diagnoses.

**FIGURE 3:**
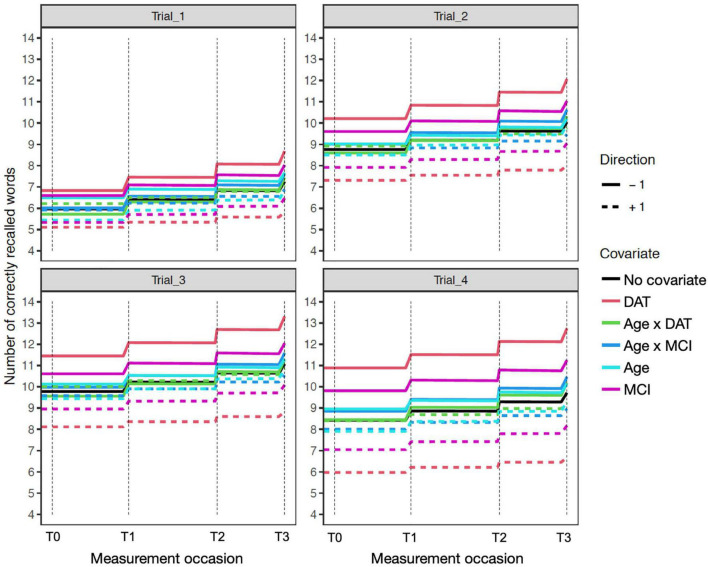
Age sensitivity model expectation plots for change in recall performance (black line) across four measurement occasions (e.g., T0: baseline, T1: 1 year). The solid and dashed colored lines depict effect modification by time independent predictors: DAT diagnosis (red lines), MCI diagnosis (magenta lines), Age (light blue lines), and interactions of Age × DAT and Age × MCI. For main effects, higher (dashed) and lower (solid) levels of the covariate modifies the level and expected patterns of change. All effects are in reference to 0 values on other covariates. The dashed red line reflects DAT, and the solid red line represents all other participants. Interaction effects only represent the total additive value of the interaction, when holding the main effects at zero.

**FIGURE 4:**
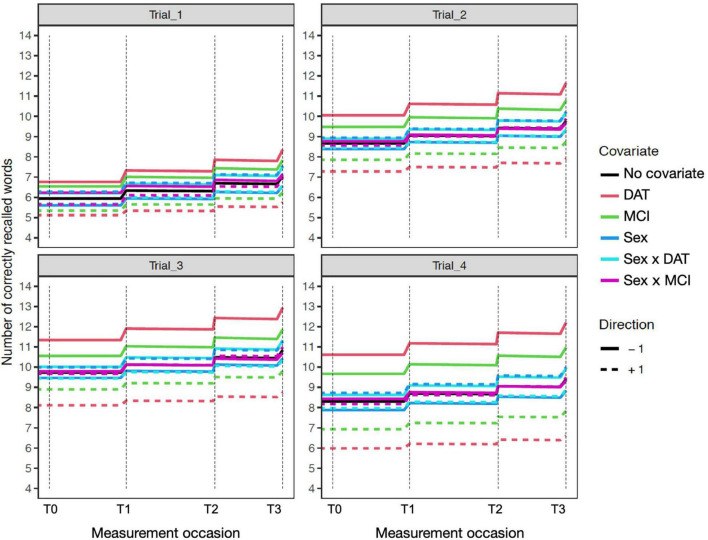
Expectation plots for change in recall performance (black line) across four measurement occasions (e.g., T0: baseline, T1: 1 year) in the sensitivity model of participant age. The solid and dashed colored lines depict effect modification by time independent predictors: DAT diagnosis (red lines), MCI diagnosis (green lines), Sex (blue lines; women: +1; men: –1), and interactions of Age × DAT and Age × MCI. For covariate effects, higher (dashed) and lower (solid) levels of the covariate are shown to modify the level and expected slope. All covariate effects are in reference to 0 values of other covariates.

The time independent predictor effects and interactions are best represented by the accompanying expectation plots (Figures 1–4). As these are likely to be unfamiliar to most readers without prior dynamic modeling experience, their interpretation benefits from some explanation. Figure 1 provides an example of the expected effects of educational attainment on performance, uncomplicated by additional interactions. The plot depicts model expectations of recall performance, measured over four occasions, with each trial type (i.e., 1–4) depicted separately in the four panels. The *y*-axes represent the number of correctly recalled words on a trial, and the *x*-axis represents time; the dashed vertical lines depict the individual measurement occasions. The black plotted line depicts the expectation of change in the total sample, in the absence of any covariates. The level of the line on the *y*-axis represents the number of words recalled for a trial in the total sample and this expected value is incrementally increased before the next measurement occasion as a function of the estimated PE parameter. Starting at baseline (T0) the line is flat until just before the second occasion (T1) where PE is first relevant. The magnitude of the increase reflects PE at that occasion. The slope of the line between T1 to T2 and from T2 to T3 also reflects the amount of positive feedback or decay in PE as estimated by positive or negative sf_Practice – the feedback component on PE that allows for increasing or reducing gains of further practice. The dashed and solid red lines show model expectations when the covariate in question is ±1 and all other covariates are zero. For dichotomous covariates like sex, this reflects group differences. Here, higher education (dashed red line) predicts higher level of performance, with stronger effects on Trials 2–4 and occasions T0–T2, but the difference is reduced at T3. The lower education group (solid red line) has a higher PE gain at the end of each occasion, despite the lower initial level. In addition, this is accompanied by greater decay (i.e., less positive practice self-feedback) on the PE process. Of note, for interactions (e.g., Figures 2–4), the dashed or solid lines represent only the interaction effect, not the interaction plus main effects. For example, in the case of Sex × DAT, the +1 line shows only the additive effect of female sex and positive DAT diagnosis, assuming the individual Sex and DAT diagnosis covariates are 0.

### Diagnostic groups model

The model with diagnostic groups as the only time independent predictor provided overall characterization of the sample (Table 3). The estimated population mean of PE was significantly positive, indicating overall improvement in performance across longitudinal occasions of 0.4 words, for the entire sample. The mean trial self-feedback (sf_Trial) parameter was very negative, implying that random changes at the trial level did not persist across occasions. In addition, the mean for the sf_Practice parameter was not significant, suggesting that gains in PE neither increase nor decrease substantially, given further repetitions. The means for the other parameters, including diffusion for each trial and total measurement error were all positive. Furthermore, the means for all four Trials were positively correlated within-subject, but there were no significant correlations with PE gain per occasion in the total sample. Evaluation of diagnostic groups as time independent predictors showed MCI and dementia diagnosis predicted lower mean performance on all four trials (Table 4), as well as a non-significant trend for DAT diagnosis predicting lower PE. Both MCI and DAT diagnoses predicted significantly higher Diffusion effects for Trial 3 only, implying that diagnoses of MCI and dementia were associated with greater random changes in Trial 3 that were nevertheless predictive of other trials, thus likely representing genuine change and not measurement error.

**TABLE 3:** Estimated population mean values and correlations for diagnostic group and sensitivity models.

	Diagnostic groups		Age		Sex		Education	
	Mean (*SD*)	95% CI	Mean (*SD*)	95% CI	Mean (*SD*)	95% CI	Mean (*SD*)	95% CI
**Population means**								
sf_Practice	0.009 (0.256)	−0.506, 0.523	−0.006 (0.244)	−0.502, 0.475	−0.049 (0.280)	−0.594, 0.504	−0.200 (0.314)	−0.824, 0.429
sf_Trial	−3.471 (1.065)	−5.671, −1.650	−3.001 (0.749)	−4.596, −1.669	−3.218 (1.051)	−5.513, −1.454	−2.733 (0.691)	−4.161, −1.545
Diffusion T1	3.795 (0.609)	2.702, 5.067	3.548 (0.445)	2.743, 4.467	3.748 (0.607)	2.688, 5.026	3.442 (0.466)	2.599, 4.432
Diffusion T2	3.523 (0.580)	2.538, 4.814	3.333 (0.431)	2.574, 4.247	3.512 (0.575)	2.487, 4.708	3.138 (0.417)	2.396, 4.009
Diffusion T3	2.781 (0.458)	2.025, 3.806	2.707 (0.352)	2.126, 3.439	2.801 (0.450)	1.993, 3.739	2.514 (0.348)	1.883, 3.244
Diffusion T4	5.499 (0.838)	4.089, 7.203	5.231 (0.650)	4.055, 6.515	4.816 (0.765)	3.449, 6.415	4.401 (0.582)	3.330, 5.583
Meas. error	0.299 (0.190)	0.080, 0.780	0.260 (0.173)	0.060, 0.713	0.233 (0.160)	0.054, 0.669	0.476 (0.923)	0.006, 2.934
Practice effect (PE)	0.399 (0.110)	0.184, 0.607	0.436 (0.117)	0.213, 0.665	0.388 (0.124)	0.145, 0.617	0.467 (0.123)	0.216, 0.714
Trial 1	6.087 (0.154)	5.781, 6.381	5.964 (0.163)	5.631, 6.263	5.939 (0.168)	5.611, 6.261	6.079 (0.161)	5.772, 6.389
Trial 2	8.782 (0.146)	8.514, 9.074	8.762 (0.149)	8.467, 9.046	8.663 (0.155)	8.366, 8.976	8.773 (0.141)	8.510, 9.056
Trial 3	9.837 (0.140)	9.578, 10.111	9.789 (0.141)	9.517, 10.055	9.733 (0.149)	9.428, 10.033	9.845 (0.136)	9.594, 10.114
Trial 4	8.478 (0.211)	8.054, 8.902	8.432 (0.212)	8.001, 8.839	8.297 (0.218)	7.877, 8.727	8.546 (0.199)	8.173, 8.925
**Population correlations**								
Trial 1–PE	0.013 (0.251)	−0.472, 0.474	−0.034 (0.224)	−0.461, 0.396	0.037 (0.315)	−0.552, 0.630	0.124 (0.331)	−0.511, 0.707
Trial 2–PE	0.186 (0.271)	−0.379, 0.681	0.102 (0.256)	−0.381, 0.588	0.199 (0.328)	−0.447, 0.766	0.293 (0.340)	−0.435, 0.811
Trial 3–PE	0.135 (0.280)	−0.463, 0.646	0.009 (0.260)	−0.495, 0.503	0.172 (0.344)	−0.505, 0.764	0.245 (0.363)	−0.502, 0.810
Trial 4–PE	0.178 (0.246)	−0.354, 0.627	0.048 (0.238)	−0.403, 0.487	0.194 (0.308)	−0.417, 0.727	0.249 (0.340)	−0.470, 0.785
Trial 2–Trial 1	0.897 (0.049)	0.783, 0.958	0.889 (0.058)	0.743, 0.967	0.901 (0.047)	0.778, 0.965	0.892 (0.057)	0.750, 0.961
Trial 3–Trial 1	0.816 (0.057)	0.686, 0.910	0.800 (0.077)	0.606, 0.911	0.824 (0.065)	0.659, 0.919	0.801 (0.076)	0.619, 0.911
Trial 4–Trial 1	0.654 (0.084)	0.476, 0.791	0.613 (0.113)	0.359, 0.798	0.634 (0.086)	0.447, 0.782	0.675 (0.099)	0.459, 0.833
Trial 3–Trial 2	0.927 (0.038)	0.832, 0.974	0.910 (0.051)	0.763, 0.972	0.925 (0.042)	0.827, 0.976	0.916 (0.045)	0.805, 0.974
Trial 4–Trial 2	0.817 (0.064)	0.674, 0.909	0.773 (0.089)	0.552, 0.901	0.799 (0.069)	0.620, 0.901	0.831 (0.076)	0.649, 0.931
Trial 4–Trial 3	0.867 (0.056)	0.724, 0.944	0.839 (0.077)	0.657, 0.942	0.843 (0.064)	0.685, 0.933	0.902 (0.055)	0.759, 0.973

95% CI, values are upper (2.5%) and lower (97.5%) bounds. sf_Practice, practice self-feedback; sf_Trial, trial self-feedback; Diffusion, standard deviation of diffusion processes for a given trial (e.g., T1 is Trial 1); Meas. error, measurement error; Trial represents manifest mean recall for each Trial, aggregated across occasions.

**TABLE 4:** Effects of diagnostic groups in the total sample.

Interaction	Mean (*SD*)	95% CI
MCI × sf_Practice	0.063 (0.095)	−0.121, 0.248
MCI × sf_Trial	0.043 (0.291)	−0.548, 0.615
MCI × Diffusion T1	−0.477 (0.330)	−1.181, 0.136
MCI × Diffusion T2	0.284 (0.264)	−0.225, 0.800
MCI × Diffusion T3	0.466 (0.217)	0.052, 0.903*
MCI × Diffusion T4	0.012 (0.397)	−0.835, 0.743
MCI × Meas. error	0.007 (0.065)	−0.114, 0.148
MCI × Practice effect	−0.120 (0.133)	−0.372, 0.157
MCI × Trial 1	−1.423 (0.218)	−1.846, −0.996*
MCI × Trial 2	−1.769 (0.210)	−2.168, −1.356*
MCI × Trial 3	−1.778 (0.204)	−2.191, −1.382*
MCI × Trial 4	−2.923 (0.310)	−3.525, −2.350*
DAT × sf_Practice	−0.002 (0.103)	−0.198, 0.200
DAT × sf_Trial	0.182 (0.312)	−0.454, 0.802
DAT × Diffusion T1	−0.047 (0.404)	−0.835, 0.716
DAT × Diffusion T2	0.404 (0.323)	−0.219, 1.071
DAT × Diffusion T3	0.730 (0.307)	0.168, 1.379*
DAT × Diffusion T4	−0.948 (0.638)	−2.227, 0.215
DAT × Meas. error	−0.013 (0.071)	−0.160, 0.113
DAT × Practice effect	−0.435 (0.257)	−0.931, 0.068+
DAT × Trial 1	−2.328 (0.303)	−2.866, −1.741*
DAT × Trial 2	−3.807 (0.297)	−4.401, −3.211*
DAT × Trial 3	−4.409 (0.295)	−4.993, −3.839*
DAT × Trial 4	−6.345 (0.410)	−7.194, −5.554*

Values are mean with standard deviation in parentheses. 95% CI, values are upper (2.5%) and lower (97.5%) bounds. sf_Practice, practice self-feedback; sf_Trial, trial self-feedback; Diffusion, standard deviation of diffusion processes for a given trial (e.g., T1 is Trial 1); Meas. error, measurement error; Trial represents manifest mean recall for each Trial, aggregated across occasions. The asterisk * denotes significant effects; the + indicates nonsignificant trends.

### Sensitivity models

Next, we evaluated independent sensitivity models to examine the modifying effects of individual differences in age, sex, and educational attainment on main effects and interactions with diagnostic group (Table 5).

**TABLE 5:** Significant and trending covariate effects and interactions with diagnostic groups in sensitivity models of age, sex and education.

Age	Mean (*SD*)	95% CI	Sex	Mean (*SD*)	95% CI	Education	Mean (*SD*	95% CI
Age × T1	−0.520 (0.152)	−0.820, −0.219	Sex × T1	0.357 (0.158)	0.038, 0.653	Educ. × T1	0.060 (0.059)	−0.053, 0.179
Age × T2	−0.255 (0.138)	−0.520, 0.007	Sex × T2	0.302 (0.136)	0.049, 0.566	Educ. × T2	0.077 (0.051)	−0.015, 0.186
Age × T3	−0.344 (0.130)	−0.614, −0.089	Sex × T3	0.280 (0.129)	0.026, 0.528	Educ. × T3	0.081 (0.048)	−0.008, 0.183
Age × T4	−0.521 (0.201)	−0.915, −0.125	Sex × T4	0.448 (0.207)	0.072, 0.841	Educ. × T4	−0.031 (0.069)	−0.167, 0.108
MCI × Diff. T1	−0.489 (0.305)	−1.129, 0.045	MCI × Diff. T1	−0.700 (0.339)	−1.445, −0.101	MCI × Diff. T1	−0.417 (0.317)	−1.052, 0.207
MCI × Diff. T2	0.389 (0.220)	−0.034, 0.778	MCI × Diff. T2	0.467 (0.220)	0.065, 0.931	MCI × Diff. T2	0.428 (0.203)	0.007, 0.818
DAT × Diff. T3	0.663 (0.281)	0.150, 1.241	DAT × Diff. T3	0.628 (0.301)	0.087, 1.283	DAT × Diff. T3	0.792 (0.271)	0.287, 1.324
DAT × Diff. T4	−1.355 (0.585)	−2.535, −0.269	DAT × Diff. T4	−0.949 (0.629)	−2.210, 0.253	DAT × Diff. T4	−1.435 (0.679)	−2.850, −0.149
DAT × PE	−0.514 (0.248)	−1.003, −0.020	DAT × PE	−0.463 (0.259)	−0.981, 0.039	DAT × PE	−0.472 (0.257)	−0.980, 0.004
Age × MCI × sf_Prac	0.020 (0.107)	−0.180, 0.231	Sex × MCI × sf_Prac	0.057 (0.101)	−0.149, 0.258	Educ. × MCI × sf_Prac	−0.097 (0.081)	−0.254, 0.063
Age × MCI × Diff. T1	0.050 (0.281)	−0.497, 0.576	Sex × MCI × Diff. T1	0.625 (0.312)	0.060, 1.273	Educ. × MCI × Diff. T1	0.178 (0.213)	−0.252, 0.554
Age × MCI × Diff. T3	−0.455 (0.219)	−0.893, −0.036	Sex × MCI × Diff. T3	0.126 (0.230)	−0.336, 0.581	Educ. × MCI × Diff. T3	0.045 (0.136)	−0.220, 0.308
Age × MCI × T1	−0.070 (0.204)	−0.441, 0.339	Sex × MCI × T1	−0.483 (0.227)	−0.925, −0.038	Educ. × MCI × T1	0.061 (0.089)	−0.114, 0.230
Age × MCI × T2	−0.385 (0.194)	−0.745, −0.019	Sex × MCI × T2	−0.167 (0.217)	−0.595, 0.256	Educ. × MCI × T2	0.040 (0.083)	−0.127, 0.197
Age × MCI × T3	−0.303 (0.186)	−0.673, 0.062	Sex × MCI × T3	−0.104 (0.207)	−0.503, 0.302	Educ. × MCI × T3	0.094 (0.081)	−0.070, 0.242
Age × MCI × T4	−0.660 (0.276)	−1.208, −0.149	Sex × MCI × T4	−0.208 (0.303)	−0.777, 0.390	Educ. × MCI × T4	0.213 (0.119)	−0.024, 0.447
Age × DAT × Diff. T4	−0.493 (0.576)	−1.623, 0.618	Sex × DAT × Diff. T4	−1.519 (0.628)	−2.800, −0.408	Educ. × DAT × Diff. T4	0.546 (0.433)	−0.328, 1.436
Age × DAT × ME	0.002 (0.058)	−0.107, 0.134	Sex × DAT × ME	0.005 (0.049)	−0.084, 0.110	Educ. × DAT × ME	0.129 (0.279)	−0.006, 0.985
Age × DAT × T1	0.549 (0.286)	−0.016, 1.130	Sex × DAT × T1	−0.646 (0.301)	−1.221, −0.048	Educ. × DAT × T1	−0.033 (0.126)	−0.276, 0.216
Age × DAT × T2	0.352 (0.265)	−0.147, 0.880	Sex × DAT × T2	−0.570 (0.283)	−1.118, −0.008	Educ. × DAT × T2	0.120 (0.119)	−0.111, 0.354
Age × DAT × T3	0.491 (0.258)	−0.010, 0.985	Sex × DAT × T3	−0.705 (0.266)	−1.240, −0.184	Educ. × DAT × T3	−0.014 (0.112)	−0.248, 0.202
Age × DAT × T4	−0.037 (0.355)	−0.729, 0.633	Sex × DAT × T4	−0.796 (0.376)	−1.499, −0.037	Educ. × DAT × T4	0.444 (0.156)	0.125, 0.752

Table depicts significant effects and interactions present in one or more of the three sensitivity models. Covariate effects that were not significant in any model are not shown. 95% CI, values are upper (2.5%) and lower (97.5%) bounds. MCI, diagnosis of amnestic or non-amnestic MCI; DAT, diagnosis of multi-domain amnestic dementia. sf_Prac, practice self-feedback; T, trial; for Diff. T1 is standard deviation of diffusion process for Trial 1; values of T1, T2, T3, T4 refer to manifest mean recall for each Trial, aggregated across occasions. PE, practice effect; ME, measurement error. Sex, men modeled as −1 and women as +1.

#### Age

The addition of years of age as a time independent predictor showed on average, older age was associated with worse performance on all four trials. However, this was qualified by interactions of mean trial performance with clinical diagnosis. Older age was associated with poorer performance on Trials 1, 2, and 3 among those with MCI diagnoses, but with trends toward better recall on trials 1 and 3 in those with DAT diagnoses (Figure 2). Moreover, the negative effect of dementia on PE gain per occasion was significant when accounting for age. A significant negative interaction of Age × MCI × Diffusion Trial 3 was due to older age attenuating the positive effects of MCI diagnosis on Trial 3 Diffusion. Here, whereas MCI diagnosis predicted higher levels of random variations in Trial 3 that benefited model prediction, this was limited by more advanced age.

#### Sex

Inclusion of participant sex in the model showed superior mean performance by women on all four recall trials. This was qualified by significant negative interactions of sex with diagnostic group predictors on Trial 1 for MCI and on all trials for DAT. As shown in the expectation plots (Figure 3), female sex was associated with lower performance in diagnosed DAT. In addition, significant positive interaction of sex with MCI on Trial 1 Diffusion, was due to higher Trial 1 Diffusion among women than men with MCI diagnoses. However, a significant negative interaction of sex with DAT on Trial 4 Diffusion showed lower predictive random changes in women than men with diagnoses of DAT on delayed recall trials.

#### Educational attainment

Greater educational attainment was marginally associated with higher performance on Trial 3 and 4 in the total sample. However, this was qualified by positive interactions between education and both MCI and DAT on Trial 4 only, where higher education predicted better delayed recall performance (Figure 4). More years of education also predicted lower measurement error in the MCI group, but higher measurement error in the DAT group.

### Subsidiary models by diagnostic groups

In a series of models specific to each diagnostic group we also evaluated separate models with the time independent predictors age, sex, and educational attainment (Table 6). Complete details of all model outputs, including population means, population correlations and effects of time independent predictor are provided in Supplementary Material.

**TABLE 6:** Significant and trending covariate effects of participant age, sex, and education on model parameters by subgroup.

	CU		MCI		DAT	
Interaction	Mean (*sd*)	95% CI	Mean (*sd*)	95% CI	Mean (*sd*)	95% CI
Age × sf_Practice	−0.002 (0.095)	−0.196, 0.175	0.044 (0.093)	−0.130, 0.217	−0.067 (0.033)	−0.130, −0.002*
Age × Diffusion T3	0.431 (0.210)	0.048, 0.909*	−0.568 (0.331)	−1.235, 0.085	−0.016 (0.360)	−0.750, 0.712
Age × Diffusion T4	0.699 (0.365)	0.034, 1.473*	−0.687 (0.409)	−1.499, 0.069	−0.060 (0.133)	−0.388, 0.138
Age × PE	0.080 (0.094)	−0.107, 0.260	−0.181 (0.131)	−0.435, 0.082	0.074 (0.201)	−0.330, 0.468
Age × Trial 1	−0.489 (0.140)	−0.763, −0.221*	−0.706 (0.139)	−0.980, −0.432*	0.163 (0.258)	−0.324, 0.627
Age × Trial 2	−0.305 (0.124)	−0.556, −0.067*	−0.660 (0.155)	−0.969, −0.375*	0.493 (0.203)	0.097, 0.893*
Age × Trial 3	−0.330 (0.104)	−0.532, −0.136*	−0.669 (0.145)	−0.972, −0.400*	0.473 (0.224)	0.047, 0.900*
Age × Trial 4	−0.482 (0.167)	−0.784, −0.175*	−1.200 (0.256)	−1.724, −0.703*	−0.090 (0.215)	−0.497, 0.313
Sex × Diffusion T3	−0.305 (0.194)	−0.693, 0.073	−0.279 (0.304)	−0.907, 0.307	−0.506 (0.348)	−1.284, 0.135
Sex × Diffusion T4	1.113 (0.322)	0.471, 1.737*	−0.019 (0.417)	−0.838, 0.785	−0.057 (0.133)	−0.403, 0.118
Education × Diffusion T4	0.753 (0.247)	0.336, 1.252*	0.305 (0.270)	−0.223, 0.852	0.450 (0.316)	0.035, 1.144+
Education × Meas. Error	−0.050 (0.010)	−0.070, −0.032*	−0.037 (0.050)	−0.123, 0.094	0.024 (0.083)	−0.033, 0.178
Education × PE	−0.018 (0.040)	−0.096, 0.062	−0.101 (0.058)	−0.217, 0.015+	−0.081 (0.080)	−0.240, 0.079
Education × Trial 1	0.035 (0.055)	−0.069, 0.146	0.107 (0.062)	−0.011, 0.227+	0.016 (0.112)	−0.205, 0.238
Education × Trial 2	0.063 (0.048)	−0.034, 0.159	0.095 (0.069)	−0.029, 0.233+	0.101 (0.094)	−0.088, 0.282
Education × Trial 3	0.072 (0.041)	−0.007, 0.153+	0.127 (0.065)	−0.002, 0.255+	0.074 (0.107)	−0.128, 0.277

Significant interactions denoted by asterisk (*). CU, cognitively unimpaired; MCI, diagnosis of amnestic or non-amnestic MCI; DAT, diagnosis of multi-domain amnestic dementia. Values are mean with standard deviation in parentheses. 95% CI, values are upper (2.5%) and lower (97.5%) bounds. sf_Practice, practice self-feedback; sf_Trial, trial self-feedback; Diffusion, standard deviation of diffusion processes for a given trial (e.g., T1 is Trial 1); Meas. error: measurement error; PE, practice effect gains; trial represents manifest mean recall for each Trial, aggregated across occasions. The + indicates nonsignificant trends.

#### Cognitively unimpaired

The three models limited to the CU participants showed significant negative correlations between PE and mean recall performance on Trials 3 and 4 (Supplementary Table 2); those with better performance in the later and delayed recall trials had lower PE gain per occasion. Older age in CU participants was associated with higher Diffusion on Trials 3 and 4, and with lower overall mean performance on all trials (Table 6). In contrast, analysis of sex differences in the CU subsample showed men have higher Trial 3 diffusion and lower Trial 4 diffusion than women. Last, the education model showed higher educational attainment was associated with higher Trial 4 Diffusion, lower measurement error, and higher Trial 3 mean recall.

#### Mild cognitive impairment

Notably, the mean estimated PE gain per occasion parameter did not differ significantly from zero in the MCI subgroup analyses (Supplementary Table 3). In addition, MCI subgroup models did not show any significant correlations between mean Trial performance and PE gain. As with the CU analysis, older age in the MCI subgroup predicted lower Diffusion on Trials 3 and 4 and lower mean performance on Trials 2, 3, and 4. Modeling effects of sex in the MCI subgroups showed women to have better recall on Trials 3 and 4. Higher educational attainment in the MCI subgroup predicted better performance on Trials 1, 2, and 3, as well as lower overall PE.

#### Dementia

The DAT subgroup models showed significantly negative PE gain estimates (Supplementary Table 4). In addition, the subgroup models for Age and Sex both produced significant negative parameter estimates for the correlations between PE and mean performance on Trial 2 and Trial 4. Older age in the DAT subgroup predicted negative sf_Practice, but better mean performance on Trials 2 and 3. Sex differences were only manifest in mean level of Trial 3, where women performed worse than men. Higher educational attainment predicted higher Trial 4 Diffusion and trends for higher measurement error and lower PE, but no apparent effects on mean Trial performance.

#### Comparison of subgroup models

The individual models by clinical diagnosis demonstrated effects that were modified by the inclusion of specific covariates, as well as those that were consistent across subgroup sensitivity models. The sf_Practice parameter appeared sensitive to clinical diagnosis, with estimates that were more negative in the CU group and closer to zero in MCI; in contrast, estimated sf_Practice was positive in the DAT subgroup, and this was magnified by older age. In addition, PE gain was negatively correlated with Trial 3 and 4 recall performance for the CU subgroup; no correlations were significant between PE gain and performance in the MCI subgroup. The dementia subgroup showed higher PE was associated with lower recall performance only on Trials 2 and 4; however, this was only manifest in sensitivity models with Age and Sex and became non-significant when accounting for differences in Education. Similarly, the manifest means for all four recall Trials were consistently positively correlated across subgroup sensitivity models for CU and MCI subgroups, whereas the dementia subgroup showed more variable patterns across sensitivity models.

Comparison of covariate effects between subgroup and sensitivity models (Table 5) shows that older age was associated with lower mean performance on all trials for CU and MCI subgroups, and with higher performance on Trials 2 and 3 in the DAT subgroup (Table 6 and Supplementary Table 5). Moreover, whereas older age predicted higher Diffusion on Trials 3 and 4 in the CU subgroup, the opposite effect was manifest for the MCI group. In addition, older age only predicted more negative sf_Practice in those with diagnosed dementia. There were fewer effects of participant sex (Supplementary Table 6), although notably, while women in the MCI subgroup had higher Trial 3 performance, this was reversed in the DAT analysis. Higher educational attainment was associated with marginal benefits on mean performance on Trials 1–3 in the CU and MCI subgroups and with higher Trial 4 Diffusion in CU and DAT subgroups, but not MCI (Supplementary Table 7). Similarly, higher education was associated with lower PE only in the MCI subgroup.

### Reparametrized to estimate within-occasion practice effects

The models reported in the present study focused on longitudinal practice effects. To address whether trial-by-trial improvements were also associated with clinical diagnosis we reparametrized the original diagnostic groups model to estimate relative within-occasion improvement. The new model estimated a parameter for Baseline performance as well as the deviations from Trial 1 for Trials 2, 3, and 4, rather than absolute performance, while otherwise maintaining the same model setup. Model results showed the population means (Supplementary Table 8) were consistent with the results from the original diagnostic groups model with one exception. The reparametrized model showed higher estimated level of Trial 3 Diffusion (mean = 4.873, sd = 0.384; 95% CI = 4.169–4.857) than the original (mean = 2.781, sd = 0.458; 95% CI = 2.025–3.806). Time independent predictor effects showed MCI and dementia diagnosis attenuated trial-by-trial improvements (Supplementary Table 9). Diagnosis did not interact with PE, measurement error, sf_Trial or sf_Practice, but both DAT and MCI diagnosis predicted significantly lower Trial 3 Diffusion. Inspection of the estimated population correlations between the trial-level deviations and parameter values for PE and Baseline performance showed higher baseline performance was associated with lower within-occasion improvement for Trial 2 and Trial 3 only (Supplementary Table 9). However, neither the trial-level deviations nor Baseline performance estimates were significantly correlated with PE. In addition, all three trial-level deviation parameters were positively correlated; greater improvement from Trial 1 tended to generalize across later trials.

## Discussion

Dynamic modeling of PE from multi-occasion verbal learning data revealed multiple notable effects associated with clinical diagnoses of MCI and dementia. First, in accord with our initial hypotheses both manifest recall performance and overall PE varied as a function of diagnostic severity. In addition to diagnosis-specific variation in levels of mean trial performance, we observed a gradient of PE across the three diagnostic groups – from positive in CU participants to significantly negative in participants diagnosed with dementia. Whereas repeated performance conferred subsequent improvements in recall for unimpaired older adults, this was not consistently the case in those with diagnosed MCI; moreover, we observed ongoing decline in participants diagnosed with DAT, despite repeated testing, as evidenced by negatively estimated PE. Notably, modeling the four recall trials as individual processes permitted estimating mean performance separately from PE. Thus, mean trial performance is modeled as a stable, trait-like factor, whereas estimated PE served as the primary measure of change. This modeling perspective contrasts with most prior efforts that model PE as a linear change within or between occasions (Duff et al., 2007; Bender et al., 2013, 2020; Goldberg et al., 2015; Gavett et al., 2016). In addition, the PE parameter does not delineate between true decline and gains due to practice, as these are not considered separable processes in a dynamic system. The sensitivity of dynamic estimates of PE and performance to clinical diagnosis demonstrates the value of dynamic modeling in longitudinal clinical aging data.

Second, the present findings revealed previously unreported relationships between clinical diagnosis and dynamic process estimates. As with PE, the sf_Practice parameter followed a gradient of positive to negative values that corresponded with diagnostic severity. Practice self-feedback provides a non-linear measure of the extent level that practice (i.e., after completing all four trials for a given occasion) can boost or reduce estimated PE at the next occasion. The more positive estimates of sf_Practice in CU participants reflects an increase in practice-related gains on subsequent occasions. In contrast, both PE and sf_Practice were negatively estimated in participants with diagnosed dementia. Thus, while recall performance declined over time in these participants even with repeated testing (i.e., as indicated by negative PE estimates), dementia diagnosis was associated with less acceleration in decline. In other words, performance appears to stabilize at a lower level above floor in those diagnosed with dementia, despite both the absence of retest improvements and overall decline. In addition, both measurement error and diffusion processes (particularly on Trials 3 and 4), were sensitive to diagnostic group and other covariate effects. The standard deviation of the diffusion processes reflects unpredictable variations in trial performance that are nevertheless useful in predicting performance on other trials. In tasks like the HVLT, recall performance on later immediate recall trials necessarily includes savings from the preceding recall trials. The reported findings suggest that meaningful variations in Trial 3 performance may provide a uniquely sensitive marker of clinical cognitive impairment and dementia. Although the interpretation of such unpredictable variations is not clear, one possibility is Trial 3 diffusion processes may partly reflect impaired executive or amnestic encoding abilities. For example, reduced mental flexibility and working memory in MCI and dementia may produce more inconsistent recall performance across study occasions. Alternatively, higher Trial 3 diffusion processes may capture increasing reliance on list recency due to impaired short-term verbal encoding ability. Nevertheless, multiple cognitive processes are potentially implicated, which are likely to be further complicated by diagnosis and etiology. Therefore, additional work relating differences in non-linear PE estimates to more fine-grained neuropsychological performance is needed to clarify the cognitive processes responsible for variations in Trial 3 diffusion or other parameter estimates.

Third, evaluation of sensitivity and subgroup models revealed important sources of individual differences that modified multiple effects and qualified several interactions. For example, older age predicted worse mean recall on all Trials in the CU and diagnosed MCI subgroups, but better recall on trials 2 and 3 among those with dementia diagnoses (Table 6). This may suggest a survivor effect, as those who reach more advanced age before onset of dementia may maintain some residual abilities that enhance recall on these trials. Age also modified trial-specific diffusion processes for CU and MCI diagnosed participants, despite positive mean Diffusion estimates in both groups (Supplementary Tables 2, 3). Whereas older age predicted higher Diffusion on Trials 3 and 4 in the CU subgroup, the opposite effect was manifest for the MCI group. This shows that in unimpaired adults older age enhances the generation of unpredictable but meaningful variation in performance but exerts the opposite effect in those diagnosed with MCI.

Greater education weakly predicted higher mean immediate recall abilities for CU and MCI. Higher educational attainment was also weakly associated with lower PE in the two subgroups with diagnoses of MCI or dementia (Table 6). Notably, estimated PE did not differ from zero in the MCI subgroup even though mean recall performance did not show a ceiling effect; furthermore, PE and recall were not significantly associated in this group. Thus, greater educational attainment in the presence of manifest cognitive impairment may predict greater loss of neurocognitive plasticity necessary to benefit from repetition. Critically, this finding should be interpreted in the context of recent reports showing education does not appear neuroprotective or to confer resilience to cognitive decline or neurodegeneration. Rather, more years of early-life education may increase premorbid level of ability and positively offset trajectories of decline (Wilson et al., 2019; Lovden et al., 2020; Nyberg et al., 2021). However, for those whose neurocognitive abilities have reached a functional threshold for impairment, higher education may be associated with accelerated declines. Here, PE appears to be a marker of such accelerated functional declines. Similarly, the advantage of female sex on tests of verbal memory (Herlitz et al., 1997; Herlitz and Rehnman, 2008; Bender et al., 2010) was largely negligible, with one notable exception. Whereas women in the MCI diagnosis subgroup had better mean recall on Trial 3, this was reversed in the more impaired participants with dementia diagnoses. As with education, it is possible that this reflects a positive offset in trajectories of decline due to higher premorbid level of verbal memory abilities, resulting in steeper declines following onset of dementia.

Under the present dynamic modeling framework, performance on each occasion reflects ongoing processes that are inherently altered by prior testing exposure or experience. Here, PE reflects total intra-person change as the combination of maintenance or decline in addition to contributions of prior experience. Therefore, while the variable gains or losses following practice are not clearly dissociable from ongoing declines, separating level from change across trials captures multiple behavioral dimensions relevant to clinical diagnosis. For example, we note that the relationship between better recall performance and lower PE was only observed in the participants with CU or dementia diagnoses, but not in those with MCI. Unimpaired participants performing closer to ceiling had less room to improve and were more likely to show reduced subsequent gains. In contrast, the negative estimates of PE in the subgroup with dementia diagnoses captures longitudinal declines – those with higher overall performance also had the furthest to decline. However, the disconnection between PE and level of performance in MCI suggests these two dimensions may provide unique diagnostic or prognostic information. This aligns with prior findings showing PE differences are a meaningful indicator of progressive decline in older adults with MCI diagnoses who exhibit low-to-moderate levels of recall performance (Duff et al., 2007; Rabin et al., 2009; Hassenstab et al., 2015; Gavett et al., 2016).

The present findings point to greater inconsistencies in responses, across trials and occasions as additional markers of cognitive impairment and dementia. We found that mean recall performance was consistently correlated across trials in the CU and MCI subgroups, but not in those diagnosed with dementia. Furthermore, correlations among Trials for the dementia subgroup showed more variable patterns across sensitivity models. In addition to declines in PE and mean recall performance, it is possible that loss of neurocognitive plasticity may also manifest as less consistent responses. Although the models were specified to focus primarily on longitudinal practice effects, such inconsistency may reflect reduced within-occasion improvement across trials. We also observed MCI and DAT diagnoses attenuated trial-by-trial improvements in the reparametrized model. Similarly, reduced short-term PE has previously been related to differences in clinical diagnosis, cognitive function, and brain structure (Duff et al., 2007, 2012; Fernandez-Ballesteros et al., 2012; Bender et al., 2020). These findings support the view that dynamic estimates of PE within and across occasions provides meaningful proxies for cognitive plasticity associated with advanced age or pathology (Baltes and Raykov, 1996; Yang, 2011). Further work is needed to identify which aspects of PE provide the most sensitive behavioral markers of ongoing declines.

### Limitations and future directions

The present findings provide important evidence regarding the value of dynamic modeling approaches in estimating longitudinal change in performance as a function of PE. The limitations in the present study methods and findings must be acknowledged, while also highlighting corresponding opportunities for further inquiry. The model treated clinical diagnosis as a time independent predictor, but this did not accurately represent the diagnostic variability manifest in 11% of the study sample across study occasions. Six participants with initial diagnoses of amnestic MCI converted to DAT, and an additional six participants originally characterized as CU later received diagnoses of MCI. In addition, 15 participants with baseline MCI diagnoses were characterized as CU at their most recent visit. Although the modeling approach used here did not attempt to account for such variation in diagnosis, further work is needed to evaluate dynamic modeling for more transient changes in cognitive status. One alteration from the present approach could be to model diagnosis as a time varying measure, provided sufficient variation is present. Similarly, data sampled more intensively or with more variable timing would also make better use of capacity for modeling time in *ctsem*. While it is possible that accounting for such variation in assessed cognitive status may affect the results, future work should examine how intra-individual variation in clinical diagnosis is manifest in HVLT recall performance and PE. Similarly, the dementia subgroup only included participants with Alzheimer’s (including mixed dementia) and including participants with other forms of dementia associated with other neurodegenerative diseases such as Lewy bodies, fronto-temporal dementia, or posterior cortical atrophy may demonstrate further sensitivity of PE and dynamic performance estimates to underlying pathologies. Furthermore, the same stimulus lists were presented on each occasion in the present study; future work should compare the effects of repeated vs. non-repeated content.

In addition, the available data for participants with dementia diagnoses was limited to three observations, although these were distributed across the actual occasions of assessment. While most statistical methods typically focus only on observed data, prior findings show patients with moderate Alzheimer’s dementia are more prone to non-response (Feng et al., 2020; Wang et al., 2021). The HVLT is a challenging task for patients with mild to moderate dementia and patients may become quickly discouraged. The modeling of non-ignorable missingness for statistical inference is a daunting task in practice owing to its unknown nature and non-identifiable model parameters. Although challenging, additional research is needed on further implementation of methods for modeling informative missingness in the context of estimating PE in a Bayesian structural equation modeling framework.

The reported findings suggest that meaningful variations in Trial 3 performance may provide a uniquely sensitive marker of clinical cognitive impairment and dementia. This may show that certain trials are more important in HVLT performance and PE, which could be useful in clinical applications. More work is needed to shed light on differences in individual trials and their potential utility in clinical applications. However, this would require substantially more individual data to generate population-based normative estimates for direct comparison with individual patient cases. Similarly, for other potential applications of these methods, such as power estimation for dementia prevention trials, a larger number of normative data would help reduce uncertainty in parameter estimates (i.e., shrink confidence intervals) for such complex dynamic models. Notably, prevention trials tend to have rigorously standardized schedules of assessment, while *ctsem* benefits from more variable timing across assessments in order to reduce uncertainty. Thus, clinical trials may benefit from increased flexibility in timing to better leverage dynamic modeling approaches. New methods for intensive behavior sampling using smartphones provide a clear opportunity to bridge this divide, as they allow for considerably more dense measurement and greater variability in timing. Future work should evaluate dynamic models of PE in large, normative data sets from acquired with such methods.

In addition, the present study only evaluated HVLT task data with 12 words per recall trial; however, the use of longer lists of 15 or 16 words in other verbal learning tasks could conceivably modify effects where CU participants performed close to ceiling. Future work should evaluate the effect of differences in cognitive load as a function of varying lengths of study lists. Similarly, the verbal nature of the data may confound lexical fluency with memory and PE; future investigations applying dynamic modeling approaches to estimate PE in non-verbal tasks and response times.

The present study generated far more testable hypotheses than it directly addressed. Nevertheless, the findings reported here demonstrate the expanded potential for evaluating new measures of performance affected by aging, neurodegeneration, or clinical diagnosis afforded by modeling non-linear dynamic processes.

### Conclusion

The present findings highlight the sensitivity of dynamically modeled estimates of PE and verbal recall to diagnosed MCI and dementia. Modeling PE as the primary measure of change of showed PE gains and non-linear practice self-feedback, as well as mean level of recall performance are sensitive to severity of cognitive impairment and clinical dementia diagnosis. Moreover, applying dynamic modeling to longitudinal verbal learning data captures new behavioral dimensions reflecting intra-individual variations that are sensitive to cognitive impairment and dementia. Dynamic modeling using the ctsem framework provides a new perspective for modeling longitudinal changes in performance due to aging and dementia.

## Data availability statement

The data analyzed in this study is subject to the following licenses/restrictions: the dataset analyzed for this study was provided as a Limited Dataset under a signed Data Use Agreement. As such it is not publicly available for analysis. This dataset will be made available to researchers only under a data-sharing agreement that provides for: (1) a commitment to using the data only for research purposes and not to attempt to identify any individual participant; (2) a commitment to securing the data using appropriate computer technology; and (3) a commitment to destroying or returning the data after analyses are completed. Requests to access these datasets should be directed to Arijit K. Bhaumik, Research Administrator, arijit@med.umich.edu.

## Ethics statement

The studies involving human participants were reviewed and approved by Michigan State University Human Research Protection Program. The patients/participants provided their written informed consent to participate in this study.

## Author contributions

BH oversaw data collection. CD developed the model in *ctsem* in consultation with AB. CD developed the code for modeling and data visualization. AG implemented and ran all models and output figures. AB interpreted model results in consultation with CD and AG. AB drafted the manuscript with assistance from MM. AG, BH, and CD contributed essential revisions. All authors approved the final version of the manuscript for submission.
