# Supplementary material for: Dynamic modeling of practice effects across the healthy aging-Alzheimer’s disease continuum
Source: Front Aging Neurosci. 2022 Jul 28;14:911559. doi: 10.3389/fnagi.2022.911559 (PMC9366308; doi:10.3389/fnagi.2022.911559)
Supplement: Supplementary file 1 [file Data_Sheet_1.PDF]

Supplementary Materials

Dynamic modeling of practice effects across the healthy aging-Alzheimer's disease continuum

Andrew R. Bender[1,2,3], Arkaprabha Ganguli[4*], Melinda Meiring[2], Benjamin M. Hampstead[3,5,6,7], Charles C. Driver[8,9]

[1]Department of Epidemiology and Biostatistics, College of Human Medicine, Michigan State University, East Lansing, Michigan, USA

[2]Graduate Program in Neuroscience, College of Natural Sciences, Michigan State University, East Lansing, Michigan, USA

[3]Michigan Alzheimer's Disease Research Center, Ann Arbor, Michigan, USA

[4]Department of Statistics and Probability, College of Natural Sciences, Michigan State University, East Lansing, Michigan, USA

[5]Mental Health Service, VA Ann Arbor Healthcare System, Ann Arbor, USA

[6]Neuropsychology Section, Department of Psychiatry, University of Michigan, Ann Arbor, USA

[7]Department of Psychology, University of Michigan, Ann Arbor, USA

[8]Institute of Education, University of Zurich, Zurich, CH

[9]Institute of Educational Evaluation, Associated Institute at the University of Zurich, Zurich, CH

*** Correspondence:**
Andrew R. Bender
arbender@msu.edu

Keywords: Practice effects, aging, learning, mild cognitive impairment, dementia, verbal memory, dynamic model, Bayesian

Complete information on the modeling approach can be found online at:

https://github.com/Bender-Lab/Practice_effects

Data analytic information

The following supplementary materials provide additional information on the data analytic procedures used. An anonymized version of the original data, R code and validation can be obtained from: https://osf.io/52s64/?view_only=5873db78acf24c13b938709c23bf8d32.

Continuous time structural equation modeling.

To analyze change, individual differences in change, and the impact of instructional interventions, we developed hierarchical Bayesian continuous time dynamic models and implemented them in the *ctsem* software (Driver and Voelkle, 2018). To account for varying observation timings and to allow for continuously interacting processes, *ctsem* estimates an underlying continuous time model, which is translated into discrete time expectations and covariance matrices by well-known approaches using matrix exponentiation (i.e., Voelkle and Oud, 2012; Voelkle, Oud, Davidov and Schmidt, 2012).

Three latent processes, including a general trend, a memory intervention effect, and a repetition effect constitute the core of the model. These processes are initialized to zero at the time of the first observation. Their evolution over time is described by the differential equation (1?), and together they predict the observed variables according to a measurement model that we describe below. The latent process differential equation is, in matrix form:

$$\frac{\mathrm{d}\boldsymbol{\eta}(t)}{\mathrm{d}t} = \mathbf{A}\boldsymbol{\eta}(t) + \mathbf{b} + \mathbf{M}\boldsymbol{\chi}(t)$$

Vector $\boldsymbol{\eta}(t)$ represents the state of the 3 latent processes as a function of time (t), and therefore $\mathrm{d}\boldsymbol{\eta}(t)/\mathrm{d}t$ simply means the gradient or directional change of the latent processes. The

diagonal matrix $\mathbf{A}$ contains freely estimated self-feedback terms of the processes, which are prefixed with sf in the variable names that appear in the output. These terms capture the rate of decline after a repetition or intervention effect, and the extent of non-linearity in the trend component. The continuous time intercept vector $\mathbf{b}$ provides a constant input to the latent processes $\boldsymbol{\eta}$, and is fixed at zero except for the trend intercept, which is fixed to 1. The time-dependent predictors, $\boldsymbol{\chi}(t)$, represent exogenous inputs, and are assumed to be zero whenever unobserved. This leads to sharp impulses that affect the system via the effect matrix $\mathbf{M}$ whenever a non-zero value on an intervention variable occurs, and these impulses then decline according to the estimated self-feedback. For identification purposes, $\mathbf{M}$ is fixed to 1 for the impact of repetitions on the repetition latent variable as well as the influence of the first instructional intervention on the memory process. The second and third instructional interventions have an estimated parameter (memInt2 and memInt3) governing the size of the impulse generated, relative to the first.

To derive expectations for discretely sampled data, the differential equation is solved and translated to a discrete time representation, for any observation $u \in \mathbf{U}$.

$$\boldsymbol{\eta}_u = \mathrm{e}^{\mathbf{A}(t_u - t_{u-1})}\boldsymbol{\eta}_{u-1} + \mathbf{A}^{-1}(\mathrm{e}^{\mathbf{A}(t_u - t_{u-1})} - \mathbf{I})\mathbf{b} + \mathbf{M}\mathbf{x}_u$$

Models also included baseline measures of age and sex, and scores produced by the confirmatory factor models of working memory and metabolic risk as covariates on all parameters of the latent process and measurement models, which were also allowed to vary across subjects as random effects. This accounts for issues such as inhomogeneity of measurement error with age and performance, and helps understand, for example, under which circumstances the strategy intervention was most effective. Covariate effect parameters are

prefixed by TIP, for time independent predictor. The resulting joint-posterior distribution for the model is:

$$p(\mathbf{\Phi}, \boldsymbol{\mu}, \mathbf{R}, \boldsymbol{\beta} | \mathbf{y}, \mathbf{x}, \mathbf{z}) \propto p(\mathbf{y} | \mathbf{\Phi}, \mathbf{x}) p(\mathbf{\Phi} | \boldsymbol{\mu}, \mathbf{R}, \boldsymbol{\beta}, \mathbf{z}) p(\boldsymbol{\mu}, \mathbf{R}, \boldsymbol{\beta})$$

Where the subject specific parameters $\mathbf{\Phi}_i$ for the system and measurement model are determined in the following manner:

$$\mathbf{\Phi}_i = \text{tform}(\boldsymbol{\mu} + \mathbf{R}\mathbf{h}_i + \boldsymbol{\beta}\mathbf{z}_i)$$

tform represents a transformation function chosen to ensure parameters are within the correct range (e.g., standard deviations must be positive). $\boldsymbol{\mu}$ parameterizes the means of the raw (i.e., before the tform operator is applied) population distributions of subject level parameters. $\mathbf{R}$ is the Cholesky factor of the raw population distribution covariance matrix, parameterizing the effect of subject specific deviations $\mathbf{h}_i$ on $\mathbf{\Phi}_i$. $\boldsymbol{\beta}$ is the raw effect of time independent predictors $\mathbf{z}_i$ on $\mathbf{\Phi}_i$.

A useful visualization of the model can be found in Figure 3, which shows the trend and memory intervention latent processes for two subjects. That figure also illustrates how the processes combine to generate predictions for specific aspects of memory performance. In this case the two forms represent predicted associative hit rate performance on the third set of items (which had no repetition), separately for two subjects. This also illustrates differences in model output when conditioning predictions based only on covariates versus also conditioning on prior observations.

Model respecification for trial-level deviations from Trial 1.

We respecified the model to obtain the subject level parameters for the deviations of Trials 2, 3, and 4 from Trial 1.

```
require(data.table)
subpars <- data.table(ctStanSubjectPars(f,cores=1)[1,,])
subpars$mm_t2diff <- subpars$mm_hvlt_t2tc - subpars$mm_hvlt_t1tc
subpars$mm_t3diff <- subpars$mm_hvlt_t3tc - subpars$mm_hvlt_t1tc
subpars$mm_t4diff <- subpars$mm_hvlt_t4tc - subpars$mm_hvlt_t1tc
cor(subpars)
```

References

Driver, C. C., & Voelkle, M. C. (2018). Hierarchical Bayesian continuous time dynamic modeling. *Psychological Methods*, *23*(4), 774.

Voelkle, M. C., & Oud, J. H. L. (2012). Continuous time modelling with individually varying time intervals for oscillating and non-oscillating processes. *British Journal of Mathematical and Statistical Psychology, 66*(1), 103-126.

Voelkle, M. C., Oud, J. H., Davidov, E., & Schmidt, P. (2012). An SEM approach to continuous time modeling of panel data: relating authoritarianism and anomia. *Psychological methods*, *17*(2), 176.

Table S1. Mean delays between measurement occasions.

Sample	T0-T1	T1-T2	T2-T3
Total	1.11 (0.24)	1.09 (0.26)	1.07 (0.15)
CN	1.17 (0.30)	1.05 (0.15)	1.02 (0.04)
MCI	1.14 (0.31)	1.14 (0.30)	1.04 (0.09)
DAT	1.05 (0.22)	1.20 (0.37)	1.39 (0.22)

Notes. Values are mean years between measurement occasions with standard deviation in parentheses. CN: cognitively normal; MCI: diagnosis of amnestic or non-amnestic MCI; DAT: diagnosis of multi-domain amnestic dementia. T0-T1: delay from baseline to 1-year follow-up.

Table S2. Sensitivity model population means and correlations for the cognitively normal subgroup

Cognitively Normal	Age		Sex		Education	
	Mean (SD)	95% CI	Mean (SD)	95% CI	Mean (SD)	95% CI
**Population means**						
sf_Practice	-0.687 (0.505)	-1.679, 0.307	-0.788 (0.502)	-1.833, 0.185	-0.725 (0.455)	-1.616, 0.139
sf_Trial	-4.757 (1.525)	-7.812, -2.015	-4.557 (0.314)	-5.165, -3.956	-4.688 (1.631)	-7.981, -1.792
Diffusion T1	4.555 (0.798)	3.129, 6.266	4.520 (0.308)	3.930, 5.152	4.580 (0.876)	3.097, 6.393
Diffusion T2	3.721 (0.664)	2.626, 5.102	3.739 (0.248)	3.256, 4.235	3.756 (0.702)	2.497, 5.227
Diffusion T3	2.878 (0.550)	1.931, 4.010	2.902 (0.232)	2.469, 3.388	2.862 (0.557)	1.896, 4.028
Diffusion T4	6.177 (1.051)	4.226, 8.438	5.167 (0.353)	4.469, 5.859	5.747 (1.078)	3.842, 8.026
Meas. Error	0.374 (0.216)	0.101, 0.954	0.357 (0.176)	0.130, 0.784	0.217 (0.103)	0.076, 0.462
PE	0.572 (0.124)	0.338, 0.800	0.552 (0.132)	0.283, 0.812	0.571 (0.121)	0.348, 0.803
Trial 1	6.401 (0.147)	6.123, 6.689	6.371 (0.166)	6.048, 6.712	6.482 (0.150)	6.201, 6.766
Trial 2	9.156 (0.128)	8.911, 9.404	9.114 (0.142)	8.843, 9.386	9.203 (0.134)	8.947, 9.469
Trial 3	10.164 (0.108)	9.963, 10.374	10.145 (0.123)	9.915, 10.391	10.233 (0.113)	10.011, 10.444
Trial 4	9.124 (0.170)	8.794, 9.452	9.130 (0.182)	8.790, 9.494	9.181 (0.173)	8.846, 9.520
**Population correlations**						
Trial 1-PE	-0.261 (0.215)	-0.609, 0.208	-0.280 (0.237)	-0.640, 0.248	-0.238 (0.217)	-0.587, 0.232
Trial 2-PE	-0.186 (0.216)	-0.531, 0.318	-0.232 (0.233)	-0.594, 0.297	-0.229 (0.214)	-0.558, 0.226
Trial 3-PE	-0.472 (0.187)	-0.747, -0.027	-0.463 (0.211)	-0.747, 0.040	-0.516 (0.184)	-0.768, -0.067
Trial 4-PE	-0.463 (0.193)	-0.743, 0.012	-0.500 (0.218)	-0.790, 0.018	-0.477 (0.199)	-0.747, 0.024
Trial 2-Trial 1	0.888 (0.058)	0.741, 0.963	0.897 (0.058)	0.755, 0.968	0.901 (0.059)	0.750, 0.973
Trial 3-Trial 1	0.786 (0.102)	0.549, 0.924	0.813 (0.097)	0.564, 0.941	0.812 (0.094)	0.584, 0.934
Trial 4-Trial 1	0.638 (0.132)	0.319, 0.838	0.653 (0.125)	0.362, 0.846	0.767 (0.102)	0.521, 0.912
Trial 3-Trial 2	0.867 (0.070)	0.700, 0.961	0.877 (0.062)	0.712, 0.960	0.884 (0.058)	0.740, 0.963
Trial 4-Trial 2	0.770 (0.092)	0.532, 0.907	0.770 (0.092)	0.553, 0.907	0.843 (0.073)	0.661, 0.944
Trial 4-Trial 3	0.819 (0.090)	0.587, 0.940	0.836 (0.078)	0.643, 0.942	0.898 (0.059)	0.741, 0.973

Note. 95% CI: values are upper (2.5%) and lower (97.5%) bounds. sf_Practice: Practice self-feedback; sf_Trial: Trial self-feedback; Diffusion: standard deviation of diffusion processes for a given trial (e.g., T1 is Trial 1); Meas. Error: measurement error; Trial represents manifest mean recall for each Trial, aggregated across occasions. PE: Practice effect gains.

Table S3. Sensitivity model population means and correlations for the diagnosed MCI subgroup

	Age		Sex		Education	
**MCI**	Mean (SD)	95% CI	Mean (SD)	95% CI	Mean (SD)	95% CI
**Population means**						
sf_Practice	0.322 (0.276)	-0.229, 0.853	0.356 (0.252)	-0.149, 0.850	0.238 (0.293)	-0.338, 0.824
sf_Trial	-4.041 (1.413)	-6.966, -1.626	-4.262 (1.928)	-8.533, -1.096	-4.332 (1.798)	-8.190, -1.283
Diffusion T1	2.780 (0.783)	1.521, 4.504	2.935 (1.015)	1.392, 5.210	2.867 (1.015)	1.301, 5.308
Diffusion T2	3.582 (0.857)	2.196, 5.421	3.846 (1.221)	1.995, 6.695	3.562 (1.160)	1.850, 6.152
Diffusion T3	2.853 (0.738)	1.665, 4.511	3.361 (1.110)	1.612, 6.016	3.071 (0.985)	1.522, 5.467
Diffusion T4	4.622 (0.990)	2.968, 6.807	4.942 (1.354)	2.752, 7.864	4.689 (1.242)	2.684, 7.558
Meas. Error	0.591 (0.256)	0.227, 1.190	0.558 (0.359)	0.156, 1.572	0.608 (0.311)	0.208, 1.366
PE	0.138 (0.119)	-0.105, 0.370	0.170 (0.123)	-0.064, 0.411	0.162 (0.135)	-0.089, 0.435
Trial 1	4.604 (0.140)	4.323, 4.874	4.698 (0.169)	4.369, 5.039	4.682 (0.151)	4.381, 4.963
Trial 2	6.941 (0.158)	6.612, 7.225	6.970 (0.177)	6.617, 7.320	7.016 (0.165)	6.682, 7.333
Trial 3	8.043 (0.144)	7.753, 8.332	8.034 (0.171)	7.682, 8.369	8.124 (0.164)	7.805, 8.432
Trial 4	5.277 (0.237)	4.820, 5.751	5.321 (0.282)	4.794, 5.854	5.454 (0.271)	4.894, 5.945
**Population correlations**						
Trial 1-PE	-0.351 (0.212)	-0.701, 0.110	-0.209 (0.213)	-0.592, 0.214	-0.138 (0.219)	-0.531, 0.300
Trial 2-PE	-0.227 (0.247)	-0.646, 0.279	0.006 (0.253)	-0.478, 0.477	-0.043 (0.224)	-0.464, 0.438
Trial 3-PE	-0.058 (0.227)	-0.455, 0.383	0.146 (0.236)	-0.341, 0.575	0.075 (0.216)	-0.305, 0.529
Trial 4-PE	0.094 (0.240)	-0.365, 0.539	0.343 (0.223)	-0.161, 0.715	0.252 (0.216)	-0.207, 0.644
Trial 2-Trial 1	0.721 (0.141)	0.379, 0.905	0.802 (0.098)	0.551, 0.931	0.791 (0.101)	0.542, 0.924
Trial 3-Trial 1	0.631 (0.142)	0.286, 0.838	0.709 (0.108)	0.466, 0.866	0.692 (0.111)	0.420, 0.855
Trial 4-Trial 1	0.298 (0.164)	-0.029, 0.613	0.498 (0.125)	0.217, 0.717	0.528 (0.130)	0.243, 0.737
Trial 3-Trial 2	0.875 (0.080)	0.664, 0.967	0.904 (0.061)	0.742, 0.977	0.913 (0.053)	0.768, 0.976
Trial 4-Trial 2	0.623 (0.145)	0.284, 0.836	0.697 (0.112)	0.443, 0.869	0.710 (0.128)	0.415, 0.891
Trial 4-Trial 3	0.777 (0.097)	0.545, 0.921	0.827 (0.071)	0.668, 0.926	0.821 (0.080)	0.605, 0.937

Note. 95% CI: values are upper (2.5%) and lower (97.5%) bounds. sf_Practice: Practice self-feedback; sf_Trial: Trial self-feedback; Diffusion: standard deviation of diffusion processes for a given trial (e.g., T1 is Trial 1); Meas. Error: measurement error; Trial represents manifest mean recall for each Trial, aggregated across occasions. PE: Practice effect gains.

Table S4. Sensitivity model population means and correlations for the diagnosed dementia subgroup

	Age		Sex		Education	
**DAT**	Mean (SD)	95% CI	Mean (SD)	95% CI	Mean (SD)	95% CI
**Population means**						
sf_Practice	0.538 (0.026)	0.486, 0.588	0.556 (0.024)	0.508, 0.603	0.431 (0.143)	0.146, 0.699
sf_Trial	-3.494 (1.836)	-7.534, -0.805	-3.166 (1.646)	-6.730, -0.631	-3.041 (2.005)	-7.531, -0.304
Diffusion T1	4.041 (1.223)	2.132, 6.959	3.943 (1.222)	1.986, 6.716	3.293 (1.247)	1.431, 6.269
Diffusion T2	3.687 (1.153)	1.933, 6.434	3.482 (1.116)	1.681, 5.914	3.540 (1.400)	1.480, 6.786
Diffusion T3	3.544 (1.193)	1.798, 6.343	3.063 (1.039)	1.461, 5.510	3.054 (1.197)	1.326, 6.043
Diffusion T4	0.565 (0.399)	0.125, 1.653	0.472 (0.375)	0.080, 1.483	1.020 (0.444)	0.428, 2.008
Meas. Error	0.099 (0.104)	0.014, 0.352	0.095 (0.084)	0.014, 0.337	0.159 (0.339)	0.006, 0.788
PE	-0.870 (0.279)	-1.408, -0.314	-0.888 (0.277)	-1.435, -0.325	-0.368 (0.203)	-0.761, 0.010
Trial 1	3.115 (0.302)	2.519, 3.715	3.139 (0.277)	2.595, 3.685	3.085 (0.281)	2.563, 3.654
Trial 2	4.248 (0.236)	3.779, 4.700	4.247 (0.240)	3.787, 4.701	4.258 (0.232)	3.807, 4.708
Trial 3	4.747 (0.274)	4.197, 5.276	4.784 (0.246)	4.320, 5.293	4.734 (0.263)	4.232, 5.264
Trial 4	0.970 (0.290)	0.398, 1.512	0.957 (0.286)	0.414, 1.530	1.080 (0.338)	0.407, 1.744
**Population correlations**						
Trial 1-PE	0.081 (0.151)	-0.216, 0.372	0.031 (0.167)	-0.301, 0.366	0.111 (0.308)	-0.494, 0.604
Trial 2-PE	-0.438 (0.145)	-0.684, -0.118	-0.367 (0.148)	-0.627, -0.053	-0.348 (0.301)	-0.785, 0.355
Trial 3-PE	-0.037 (0.144)	-0.318, 0.256	-0.018 (0.137)	-0.278, 0.250	-0.094 (0.333)	-0.667, 0.456
Trial 4-PE	-0.964 (0.016)	-0.983, -0.930	-0.967 (0.012)	-0.984, -0.938	-0.584 (0.406)	-0.947, 0.573
Trial 2-Trial 1	0.390 (0.228)	-0.133, 0.744	0.358 (0.253)	-0.180, 0.757	0.460 (0.220)	-0.021, 0.825
Trial 3-Trial 1	0.550 (0.187)	0.103, 0.835	0.484 (0.216)	0.003, 0.826	0.581 (0.214)	0.107, 0.892
Trial 4-Trial 1	0.043 (0.156)	-0.266, 0.339	0.062 (0.161)	-0.267, 0.370	0.178 (0.202)	-0.224, 0.551
Trial 3-Trial 2	0.764 (0.139)	0.414, 0.929	0.803 (0.114)	0.517, 0.944	0.775 (0.129)	0.440, 0.940
Trial 4-Trial 2	0.534 (0.147)	0.204, 0.770	0.455 (0.137)	0.170, 0.691	0.480 (0.235)	-0.109, 0.817
Trial 4-Trial 3	0.155 (0.148)	-0.135, 0.431	0.124 (0.135)	-0.139, 0.377	0.155 (0.241)	-0.438, 0.524

Note. 95% CI: values are upper (2.5%) and lower (97.5%) bounds. sf_Practice: Practice self-feedback; sf_Trial: Trial self-feedback; Diffusion: standard deviation of diffusion processes for a given trial (e.g., T1 is Trial 1); Meas. Error: measurement error; Trial represents manifest mean recall for each Trial, aggregated across occasions. PE: Practice effect gains.

Table S5. Covariate effects of Age on model parameters by subgroup.

	CN		MCI		DAT	
Interaction	Mean (sd)	95% CI	Mean (sd)	95% CI	Mean (sd)	95% CI
Age × sf_Practice	-0.002 (0.095)	-0.196, 0.175	0.044 (0.093)	-0.130, 0.217	-0.067 (0.033)	-0.130, -0.002 *
Age × sf_Trial	0.160 (0.317)	-0.477, 0.758	-0.199 (0.307)	-0.877, 0.393	0.065 (0.293)	-0.532, 0.646
Age × Diffusion T1	-0.065 (0.258)	-0.549, 0.465	0.002 (0.300)	-0.548, 0.597	0.085 (0.437)	-0.817, 0.981
Age × Diffusion T2	0.060 (0.229)	-0.358, 0.538	-0.207 (0.341)	-0.879, 0.488	0.463 (0.347)	-0.130, 1.241
Age × Diffusion T3	0.431 (0.210)	0.048, 0.909 *	-0.568 (0.331)	-1.235, 0.085	-0.016 (0.360)	-0.750, 0.712
Age × Diffusion T4	0.699 (0.365)	0.034, 1.473 *	-0.687 (0.409)	-1.499, 0.069	-0.060 (0.133)	-0.388, 0.138
Age × Meas. Error	0.023 (0.065)	-0.088, 0.173	0.047 (0.098)	-0.166, 0.243	0.001 (0.033)	-0.056, 0.061
Age × PE	0.080 (0.094)	-0.107, 0.260	-0.181 (0.131)	-0.435, 0.082	0.074 (0.201)	-0.330, 0.468
Age × Trial 1	-0.489 (0.140)	-0.763, -0.221 *	-0.706 (0.139)	-0.980, -0.432 *	0.163 (0.258)	-0.324, 0.627
Age × Trial 2	-0.305 (0.124)	-0.556, -0.067 *	-0.660 (0.155)	-0.969, -0.375 *	0.493 (0.203)	0.097, 0.893 *
Age × Trial 3	-0.330 (0.104)	-0.532, -0.136 *	-0.669 (0.145)	-0.972, -0.400 *	0.473 (0.224)	0.047, 0.900 *
Age × Trial 4	-0.482 (0.167)	-0.784, -0.175 *	-1.200 (0.256)	-1.724, -0.703 *	-0.090 (0.215)	-0.497, 0.313

Note. CN: cognitively normal; MCI: diagnosis of amnestic or non-amnestic MCI; DAT: diagnosis of dementia of the Alzheimer's type. 95% CI: values are upper (2.5%) and lower (97.5%) bounds. sf_Practice: Practice self-feedback; sf_Trial: Trial self-feedback; Diffusion: standard deviation of diffusion processes for a given trial (e.g., T1 is Trial 1); Meas. Error: measurement error; Trial represents manifest mean recall for each Trial, aggregated across occasions. PE: Practice effect gains.

Table S6. Covariate effects of participant sex on model parameters by subgroup.

	CN		MCI		DAT	
Interaction	Mean (sd)	95% CI	Mean (sd)	95% CI	Mean (sd)	95% CI
Sex × sf_Practice	0.002 (0.096)	-0.186, 0.177	0.031 (0.091)	-0.152, 0.214	-0.026 (0.024)	-0.072, 0.020
Sex × sf_Trial	0.009 (0.320)	-0.626, 0.626	-0.032 (0.322)	-0.636, 0.607	-0.146 (0.275)	-0.698, 0.389
Sex × Diffusion T1	-0.086 (0.266)	-0.610, 0.432	0.305 (0.283)	-0.210, 0.917	0.151 (0.372)	-0.599, 0.900
Sex × Diffusion T2	-0.156 (0.237)	-0.647, 0.294	-0.160 (0.325)	-0.872, 0.449	-0.298 (0.330)	-0.958, 0.279
Sex × Diffusion T3	-0.305 (0.194)	-0.693, 0.073	-0.279 (0.304)	-0.907, 0.307	-0.506 (0.348)	-1.284, 0.135
Sex × Diffusion T4	1.113 (0.322)	0.471, 1.737 *	-0.019 (0.417)	-0.838, 0.785	-0.057 (0.133)	-0.403, 0.118
Sex × Meas. Error	0.003 (0.064)	-0.127, 0.119	0.009 (0.113)	-0.220, 0.276	0.000 (0.024)	-0.049, 0.046
Sex × PE	0.058 (0.109)	-0.158, 0.258	-0.049 (0.117)	-0.279, 0.180	0.033 (0.235)	-0.432, 0.470
Sex × Trial 1	0.242 (0.155)	-0.077, 0.536	0.056 (0.157)	-0.249, 0.359	-0.298 (0.258)	-0.789, 0.212
Sex × Trial 2	0.191 (0.139)	-0.082, 0.453	0.230 (0.175)	-0.100, 0.569	-0.231 (0.229)	-0.682, 0.218
Sex × Trial 3	0.146 (0.121)	-0.095, 0.382	0.347 (0.169)	0.014, 0.691 *	-0.659 (0.240)	-1.166, -0.226 *
Sex × Trial 4	0.133 (0.176)	-0.223, 0.481	0.455 (0.274)	-0.048, 0.970	-0.059 (0.248)	-0.545, 0.439

Note. CN: cognitively normal; MCI: diagnosis of amnestic or non-amnestic MCI; DAT: diagnosis of dementia of the Alzheimer's type. 95% CI: values are upper (2.5%) and lower (97.5%) bounds. sf_Practice: Practice self-feedback; sf_Trial: Trial self-feedback; Diffusion: standard deviation of diffusion processes for a given trial (e.g., T1 is Trial 1); Meas. Error: measurement error; Trial represents manifest mean recall for each Trial, aggregated across occasions. PE: Practice effect gains.

Table S7. Covariate effects of participant educational attainment on model parameters by subgroup.

	CN		MCI		DAT	
Interaction	Mean (sd)	95% CI	Mean (sd)	95% CI	Mean (sd)	95% CI
Education × sf_Practice	0.056 (0.092)	-0.121, 0.227	0.026 (0.041)	-0.053, 0.109	0.033 (0.049)	-0.066, 0.126
Education × sf_Trial	0.027 (0.250)	-0.470, 0.529	0.150 (0.301)	-0.407, 0.725	0.086 (0.254)	-0.475, 0.550
Education × Diffusion T1	0.098 (0.159)	-0.204, 0.439	0.120 (0.201)	-0.310, 0.502	0.076 (0.350)	-0.468, 0.900
Education × Diffusion T2	0.107 (0.133)	-0.131, 0.385	0.268 (0.227)	-0.172, 0.739	0.204 (0.292)	-0.230, 0.886
Education × Diffusion T3	0.075 (0.113)	-0.157, 0.291	0.030 (0.176)	-0.345, 0.366	0.063 (0.260)	-0.332, 0.645
Education × Diffusion T4	0.753 (0.247)	0.336, 1.252 *	0.305 (0.270)	-0.223, 0.852	0.450 (0.316)	0.035, 1.144 +
Education × Meas. Error	-0.050 (0.010)	-0.070, -0.032 *	-0.037 (0.050)	-0.123, 0.094	0.024 (0.083)	-0.033, 0.178
Education × PE	-0.018 (0.040)	-0.096, 0.062	-0.101 (0.058)	-0.217, 0.015 +	-0.081 (0.080)	-0.240, 0.079
Education × Trial 1	0.035 (0.055)	-0.069, 0.146	0.107 (0.062)	-0.011, 0.227 +	0.016 (0.112)	-0.205, 0.238
Education × Trial 2	0.063 (0.048)	-0.034, 0.159	0.095 (0.069)	-0.029, 0.233 +	0.101 (0.094)	-0.088, 0.282
Education × Trial 3	0.072 (0.041)	-0.007, 0.153 +	0.127 (0.065)	-0.002, 0.255 +	0.074 (0.107)	-0.128, 0.277
Education × Trial 4	-0.004 (0.059)	-0.117, 0.110	0.077 (0.111)	-0.130, 0.291	0.150 (0.144)	-0.129, 0.427

Note. CN: cognitively normal; MCI: diagnosis of amnestic or non-amnestic MCI; DAT: diagnosis of dementia of the Alzheimer's type. 95% CI: values are upper (2.5%) and lower (97.5%) bounds. sf_Practice: Practice self-feedback; sf_Trial: Trial self-feedback; Diffusion: standard deviation of diffusion processes for a given trial (e.g., T1 is Trial 1); Meas. Error: measurement error; Trial represents manifest mean recall for each Trial, aggregated across occasions. PE: Practice effect gains.

Table S8. Population means for the reparametrized model of trial-level deviations

Population means	Mean (SD)	95% CI
sf_Practice	0.098 (0.277)	-0.430, 0.096
sf_Trial	-3.065 (0.791)	-4.684, -3.043
Diffusion T1	3.588 (0.553)	2.620, 3.551
Diffusion T2	3.243 (0.541)	2.344, 3.218
Diffusion T3	4.873 (0.384)	4.169, 4.857
Diffusion T4	5.119 (0.709)	3.8845, 5.103
Meas. Error	0.506 (0.372)	0.107, 0.407
PE	0.302 (0.100)	0.106, 0.302
Baseline	6.479 (0.148)	6.170, 6.481
T2Δ	2.671 (0.092)	2.491, 2.669
T3Δ	3.699 (0.105)	3.497, 3.696
T4Δ	2.493 (0.170)	2.158, 2.491

Note. These results are from the reparametrized model that included the Trial level deviations from Trial 1 (e.g., T2Δ is the difference between Trial 2 and Trial 1). 95% CI: values are upper (2.5%) and lower (97.5%) bounds. sf_Practice: Practice self-feedback; sf_Trial: Trial self-feedback; Diffusion: standard deviation of diffusion processes for a given trial (e.g., T1 is Trial 1); Meas. Error: measurement error; Trial represents manifest mean recall for each Trial, aggregated across occasions. PE: Practice effect gains; Baseline: aggregate performance at first assessment.

Table S9. Dynamic covariate effects from model reparametrized to estimate trial-level deviations.

Interaction	Mean (sd)	95% CI
MCI × sf_Practice	0.058 (0.097)	-0.145, 0.238
MCI × sf_Trial	-0.008 (0.283)	-0.563, 0.565
MCI × Diffusion T3	-0.525 (0.218)	-0.931, -0.096 *
MCI × Meas. Error	-0.020 (0.114)	-0.313, 0.179
MCI × PE	-0.009 (0.119)	-0.244, 0.229
MCI × Baseline	-1.954 (0.226)	-2.381, -1.492 *
MCI × T2Δ	-0.329 (0.152)	-0.614, -0.023 *
MCI × T3Δ	-0.298 (0.167)	-0.621, 0.030
MCI × T4Δ	-1.664 (0.258)	-2.169, -1.161 *
DAT × sf_Practice	-0.005 (0.103)	-0.208, 0.197
DAT × sf_Trial	0.112 (0.289)	-0.441, 0.672
DAT × Diffusion T3	-0.857 (0.316)	-1.494, -0.249 *
DAT × Meas. Error	0.001 (0.105)	-0.232, 0.241
DAT × PE	-0.113 (0.236)	-0.569, 0.375
DAT × Baseline	-3.630 (0.304)	-4.215, -3.031 *
DAT × T2Δ	-1.347 (0.241)	-1.837, -0.878 *
DAT × T3Δ	-1.865 (0.255)	-2.355, -1.381 *
DAT × T4Δ	-4.154 (0.351)	-4.806, -3.474 *

Note. These results are from the reparametrized model that included the Trial level deviations from Trial 1 (e.g., T2Δ is the difference between Trial 2 and Trial 1). MCI: diagnosis of amnestic or non-amnestic mild cognitive impairment; DAT: diagnosis of dementia of the Alzheimer's type. 95% CI: values are upper (2.5%) and lower (97.5%) bounds. sf_Practice: Practice self-feedback; sf_Trial: Trial self-feedback; Diffusion: standard deviation of diffusion processes for a given trial (e.g., T1 is Trial 1); Meas. Error: measurement error; PE: Practice effect gains; Baseline: mean estimate for first trial recall performance. Only effects that were significant or included PE or nonlinear estimates are shown.

Table S10. Population correlations between trial-level deviations and dynamic parameter estimates.

Interaction	Mean (sd)	95% CI
T2Δ–Baseline	-0.4696 (0.1733)	-0.7295, -0.0475
T3Δ–Baseline	-0.4348 (0.1532)	-0.6763, -0.0805
T4Δ–Baseline	-0.0754 (0.1523)	-0.3673, 0.2428
Baseline–PE	-0.0204 (0.235)	-0.4516, 0.4297
T2Δ–PE	0.4249 (0.2619)	-0.1986, 0.821
T3Δ–PE	0.2258 (0.2648)	-0.3063, 0.6807
T4Δ–PE	0.2618 (0.218)	-0.1859, 0.645
T2Δ–T3Δ	0.7828 (0.1508)	0.3515, 0.9533
T2Δ–T4Δ	0.6551 (0.1663)	0.256, 0.883
T3Δ–T4Δ	0.7014 (0.1419)	0.3548, 0.8959

Note. The Δ symbol denotes estimated Trial-level deviation scores, or the differences in performance between Trial 1 and Trial 2 (T2Δ), Trial 3 (T3Δ), and Trial 4 (T4Δ) in the reparametrized model. Values are means with standard deviations (sd); 95% CI: values are upper (2.5%) and lower (97.5%) bounds. PE: practice effects. Baseline estimates reflect overall performance at the first occasion. Here, higher baseline performance is associated with lower within-occasion improvement across recall trials, but not PE. However, greater trial-level improvement is significantly correlated across trials.
